# *Helicobacter cinaedi* is a human-adapted lineage in the *Helicobacter cinaedi/canicola/‘magdeburgensis*’ complex

**DOI:** 10.1099/mgen.0.000830

**Published:** 2022-05-10

**Authors:** Yasuhiro Gotoh, Yuya Atsuta, Takako Taniguchi, Ruriko Nishida, Keiji Nakamura, Yoshitoshi Ogura, Naoaki Misawa, Tetsuya Hayashi

**Affiliations:** ^1^​ Department of Bacteriology, Faculty of Medical Sciences, Kyushu University, Fukuoka, Japan; ^2^​ Department of Hematology, Tokyo Metropolitan Cancer and Infectious Diseases Center Komagome Hospital, Tokyo, Japan; ^3^​ Center for Animal Disease Control, University of Miyazaki, Miyazaki, Japan; ^4^​ Division of Microbiology, Department of Infectious Medicine, Kurume University School of Medicine, Kurume, Fukuoka, Japan

**Keywords:** *Helicobacter cinaedi*, *Helicobacter canicola*, enterohepatic *Helicobacter*, genome comparison, human adaptation, Type VI secretion system, metagenome data search

## Abstract

*
Helicobacter cinaedi* is an enterohepatic *Helicobacter* that causes bacteremia and other diseases in humans. While *H. cinaedi *-like strains are isolated from animals, including dog isolates belonging to a recently proposed *H. canicola *, little is known about the genetic differences between *H. cinaedi* and these animal isolates. Here, we sequenced 43 *H. cinaedi-* or *H. canicola *-like strains isolated from humans, hamsters, rats and dogs and collected 81 genome sequences of *H. cinaedi *, *H. canicola* and other enterohepatic *Helicobacter* strains from public databases. Genomic comparison of these strains identified four distinct clades (clades I–IV) in *H. cinaedi/canicola/*‘*magderbugensis*’ (HCCM) complex. Among these, clade I corresponds to *H. cinaedi sensu stricto* and represents a human-adapted lineage in the complex. We identified several genomic features unique to clade I. They include the accumulation of antimicrobial resistance-related mutations that reflects the human association of clade I and the larger genome size and the presence of a CRISPR-Cas system and multiple toxin-antitoxin and restriction-modification systems, both of which indicate the contribution of horizontal gene transfer to the evolution of clade I. In addition, nearly all clade I strains but only a few strains belonging to one minor clade contained a highly variable genomic region encoding a type VI secretion system (T6SS), which could play important roles in gut colonization by killing competitors or inhibiting their growth. We also developed a method to systematically search for *H. cinaedi* sequences in large metagenome data sets based on the results of genome comparison. Using this method, we successfully identified multiple HCCM complex-containing human faecal metagenome samples and obtained the sequence information covering almost the entire genome of each strain. Importantly, all were clade I strains, supporting our conclusion that *H. cinaedi sensu stricto* is a human-adapted lineage in the HCCM complex.

## Data Summary

The raw read sequences and complete genome sequences obtained in this study were deposited in GenBank/EMBL/DDBJ under the BioProject accession number PRJDB12330.

**Impact Statement**

*
Helicobacter cinaedi* causes bacteremia and other diseases in both immunocompromised and immunocompetent hosts. While *H. cinaedi *-like strains are isolated from animals, little is known about the genetic differences between *H. cinaedi* and these animal isolates. This study compared the genome sequences of strains belonging to the *H. cinaedi/canicola/*‘*magdeburgensis*’ (HCCM) complex and identified four distinct clades, I–IV. Clade I corresponds to *H. cinaedi sensu stricto* and apparently represents a human-adapted lineage in the complex, which has several characteristic genomic features, such as the accumulation of antimicrobial resistance-related mutation and highly variable genomic regions encoding a type VI secretion system. A method to systematically search for *H. cinaedi* sequences in human gut metagenome sequences was also developed, and clade I strain-containing samples were successfully identified in healthy individuals and colorectal cancer patients along with the sequences covering nearly entire genomes of these strains. These data provide an important basis for reclassification of the HCCM complex and novel insights into the evolution and host adaptation of *H. cinaedi sensu stricto*.

## Introduction

*Helicobacter cinaedi* is a spiral-shaped Gram-negative rod species belonging to a group of *Helicobacter* species called enterohepatic *Helicobacter *. Although this micro-organism was first isolated in the USA from an HIV-positive, homosexual man suffering from proctitis and enteritis [1], it causes infections even in immunocompetent individuals. While *H. cinaedi* causes not only gastroenteritis and proctitis but also bacteremia, erysipelas, cellulitis, arthritis and neonatal meningitis [2, 3], most isolates have been obtained from blood cultures and bacterial translocation was previously shown to be one of the routes of *H. cinaedi* bacteremia [4]. *H. cinaedi* also causes nosocomial transmission, in which asymptomatic carriers are involved [5]. As frequent recurrence is an additional feature of *H. cinaedi* infection, long-term antibiotic eradication has been proposed [2], but guidelines for antibiotic therapy have not yet been established.

Isolation of *H. cinaedi* from a variety of animals, such as dogs and hamsters, has also been reported, suggesting a possibility of zoonosis. However, there are no reliable epidemiological data to indicate transmission from these animals to human patients. The classification and species identification of this organism has also been problematic because (i) it is difficult to identify the species by biochemical properties; (ii) ATCC 35863, which was initially proposed as the type strain of *H. cinaedi *, was later reclassified as *H. fennelliae* [6]; and (iii) misidentification as *H. westmeadii* or undefined species occurs in 16S rRNA sequence-based species identification [6]. Currently, *cdtB* and *gyrB* are often used as genetic markers for the identification of *H. cinaedi* [7], and the use of MALDI-TOF MS has also been reported [8, 9]. Whole-genome sequencing (WGS)-based identification or classification has also been applied to *H. cinaedi *, and strains isolated from dogs were recently proposed as a new species, *H. canicola *, based on genome sequence homology [10]. While there are several reports on WGS analyses of human isolates, including the complete sequence determination of strains PAGU611 [11] and ATCC BAA-847^T^ [12] and screens to identify mutations responsible for antimicrobial resistance (AMR) [13] and nosocomial infections [5], WGS analyses of isolates from nonhuman sources are limited to the abovementioned report on *H. canicola* [10]. As a detailed comparison of WGS between *H. cinaedi sensu stricto* and *H. canicola* has not yet been performed, their genetic differences have yet to be elucidated. Little is also known about the factors related to *H. cinaedi* pathogenesis.

The aim of this study was to determine the population structure and genetic differences of *H. cinaedi *, *H. canicola* and ‘*H. magdeburgensis*’, a close relative of these two species [referred to as the *H. cinaedi */*canicola*/‘*magdeburgensis*’ (HCCM) complex in this study]. We sequenced 43* H. cinaedi-* or *H. canicola *-like strains isolated from humans, hamsters, rats and dogs and collected 81 genome sequences of *H. cinaedi *, *H. canicola* and other enterohepatic *Helicobacter* strains from public databases. Genomic comparison of these strains identified four distinct clades in the HCCM complex. We found that clade I corresponds to *H. cinaedi sensu stricto* and represents a human-adapted lineage in the HCCM complex, and identified several characteristic genomic features of clade I. Furthermore, we developed a method to systematically search for *H. cinaedi* sequences in large human gut metagenome sequence data sets and successfully identified clade I genomes in multiple metagenome sequences.

## Methods

### Bacterial strains and DNA sequencing

The lists of all strains analysed in this study are provided in Tables S1 and S2 (available in the online version of this article). Of the 43 strains sequenced in this study, 15 were isolated from blood cultures in routine clinical investigations using the BacT/Alert (Biomérieux, Japan) or BACTEC 9050 blood culture (BD Bioscience, Japan) system in Kyushu University Hospital between 2009 and 2017. Three hamster (T34, T35 and T36) and seven dog isolates (94105, N52, T1, T2, T3, T13 and T22) were described previously [8, 14]. The remaining 17 strains were obtained from the culture collection at the University of Göteborg (CCUG, Sweden), the American Type Culture Collection (ATCC, USA), and the National Collection of Type Cultures (NCTC, England). Genomic DNA was purified using the DNeasy Blood and Tissue Kit (Qiagen, Hilden, Germany) from bacterial cells cultured for 3–5 days in 10 ml of Brucella broth under microaerobic conditions created using the MicroAero gas generator and Hydrogen gas generator (Mitsubishi Gas Chemical, Japan). Genomic DNA libraries were prepared using the NEBNext Ultra II FS DNA Library Preparation Kit (New England Biolabs, MA, USA) and sequenced on the Illumina MiSeq platform to generate paired-end sequence reads (301 bp ×2). The complete genome sequences of eight strains and Illumina sequence reads of 15 strains, which were deposited as *H. cinaedi* or *H. canicola *, were downloaded from the NCBI Genome and SRA databases, respectively. Genome sequences of enterohepatic *Helicobacter* species used in this study were also obtained from the NCBI genome database.

Genome assembly of Illumina sequence reads was performed using platanus_B ver. 1.2.2 [15]. The quality of genome sequences was assessed using CheckM ver. 1.1.2 [16] with ≥98 % completeness and ≤2 % contamination to remove low-quality sequences. To obtain the complete sequence of strain 94 105, paired-end sequence reads were assembled using platanus_B, and gaps were closed by PCR amplification and capillary sequencing of PCR products. For the sequencing of strain T36, genomic DNA was purified using Genomic-tip 100/G (Qiagen). A long-read sequencing library was prepared with the Rapid Barcoding Kit (SQK-RBK004), sequenced using the R9.4.1 flow cell on the Oxford Nanopore Technologies (ONT) MinION platform and base-called using Guppy GPU ver. 3.4.5 (ONT). Hybrid assembly with the combination of long reads and Illumina short reads was performed using Unicycler ver. 0.4.8 [17], followed by error correction using pilon ver. 1.23 [18] with short and long reads.

### Genome sequence analyses

Gene annotation was performed using dfast ver. 1.2.6 [19] with the parameter ‘--use_genemarks2 bact’. Average nucleotide identity (ANI) values were calculated using pyani ver. 0.2.10 [20] and visualized in R with the pheatmap package. Pangenome analysis was performed using Roary ver. 1.2.3 [21] with the parameter ‘-i 80 cd 99 s’. Based on the sequences of the core genes identified by Roary, phylogenetic trees were constructed using RaxML-NG ver. 1.0.1 [22] with the best model inferred by ModelTest-NG ver. 0.1.6 [23] and 100 bootstrap replicates. Genetic population structure was inferred using hierBAPS [24]. The following two classes of genes were identified based on the Roary output: (i) genes that were present in more than 80 % of the strains in clade I and less than 20 % of the non-clade I strains and (ii) genes that were present in less than 20 % of the clade I strains and more than 80 % of the non-clade I strains. GenomeMatcher ver. 3.0.2 [25] was used for sequence comparison and visualization of the results.

### Analyses of antimicrobial resistance (AMR)-related mutations, AMR genes, antimicrobial susceptibility test and type VI secretion system (T6SS) genes, CRISPR-Cas typing and sequence typing

The presence of eight known AMR-related point mutations [5, 13, 26] (two in *pbpA*, two in *ftsI*, two in *gyrA*, one in *gyrB* and one in 23S ribosomal RNA) in each genome was investigated using Clustal Omega ver. 1.2.4 [27]. Horizontally acquired AMR genes were searched using NCBI AMRFinderPlus ver. 3.6.10 [28] with the 2020.3.20.1 database. Antimicrobial-susceptibility testing was conducted using the agar dilution method as described previously [5]. The TXSSscan database [29] was used to annotate T6SS component genes. To identify *tssI* genes, we searched each genome by tblastn ver. 2.10.1 using the TssI sequences detected by TXSSscan as queries (identity ≥90 % and query coverage ≥50 %). CRISPR-Cas genes were predicted and classified by using CRISPRCasTyper ver. 1.6.0 [30]. Sequence types (STs) of strains were determined using mlst ver. 2.19 (https://github.com/tseemann/mlst) with the *H. cinaedi* multilocus sequence typing (MLST) scheme of PubMLST [31].

### Analyses of plasmids and prophages

The distribution of seven plasmids identified in strain T36 was determined by searching for the sequences encoding the replication initiation (Rep) protein homologues of each plasmid using tblastn ver. 2.10.1 with thresholds of 95 % amino acid identity and 60 % query coverage. Platon ver. 1.5.8 [32] and plasmidVerify (https://github.com/ablab/plasmidVerify) [33] were used for plasmid search in draft genomes. Prophage search was performed using PhiSpy ver. 4.1.0 [34].

### Phylogenetic analyses of the region encoding nine T6SS core genes

Sequences of the genome region from *tssG* to *tssL* of eight clade I finished genomes and two clade II and one clade III genomes that contained these T6SS genes were aligned by Clustal Omega ver. 1.2.4 and potential recombinogenic sequences (a 156 bp sequence in *tssL* and a 715 bp sequence of *tssA*- and *tssJ*-encoding region) were removed by Gubbins ver. 3.2.0. Based on the obtained sequence alignment (8215 bp in length), a phylogenetic tree was constructed using RaxML-NG ver.1.0.1 and 1000 bootstrap replicates with the TIM1ef model.

### Analyses of the *H. cinaedi *-derived sequences in faecal metagenomic data

Based on the pangenome analysis of the 67 HCCM strains, we identified the genes that were conserved in all clade I strains but absent in all non-clade I strains (seven genes, listed in Table S3) and the genes that were conserved in all HCCM complex strains examined (526 genes, Table S3). In three genes selected from the former group of genes, three 31-mer probe sequences were designed to specifically detect clade I strains (Table S4). Three genes were also selected from the latter group of genes and another set of three 31-mer sequences was designed to detect strains belonging to the entire HCCM complex (Table S4). The genes used for probe design were selected with the following criteria: (i) single copy gene, (ii) all members in the gene cluster have the same length, and (iii) greater than 300 bp. Faecal metagenomic sequence data analysed were selected by key-word search using ‘txid408170 and RANDOM[Selection] and country name’ in the NCBI SRA database (https://www.ncbi.nlm.nih.gov/sra). In total, 715 SRA datasets from 22 BioProjects were analysed (Table S5). SRA data that had three or more hits with two probe sets were defined as positive results. To select the complete *H. cinaedi* genome sequences used as references in genome-wide mapping analysis, we mapped SRA data to the sequence of seven loci that are used in *H. cinaedi* MLST scheme in PubMLST using the BWA-MEM algorithm in bwa ver. 0.7.17 [35] to identify the ST of the strain present in the data and used the complete genome sequence of a *H. cinaedi* strain belonging to the same ST as the reference. Finally, we mapped all short reads in the SRA data to the reference with thresholds of sequence identity ≥97 % and coverage ≥90 % and calculated the sequence coverage using CoverM ver. 0.4.0 (window size: 1 kb) (https://github.com/wwood/CoverM).

### Statistical analysis

Statistical analyses were conducted using the ggplot2 package ver. 3.3.5 [36] in the R software ver. 3.6.3 [37]. The *P*-values less than 0.05 were considered statistically significant.

## Results

### Strain set of HCCM complex

The 67 HCCM complex strains analysed in this study were isolated between 1983 and 2017 from the blood (*n*=40) and faeces (*n*=6) of humans and the faeces of hamsters (*n*=3), rats (*n*=1), mice (*n*=1), dogs (*n*=13) and unknown sources (*n*=3) in ten countries (Table S1). Finished genome sequences of eight human blood isolates were obtained from the NCBI database, and those of a hamster strain (T36) and a dog strain (94105) [14] were determined in this study. Draft genome sequences of 43 strains were determined in this study, and those of seven isolates were obtained by assembling sequence reads available in the NCBI SRA database.

### ANI-based clustering of the HCCM complex

To understand the phylogenetic relationships of the HCCM complex with the other enterohepatic *Helicobacter* species, we calculated pairwise ANI values between the 67 HCCM complex strains and 57 strains belonging to the other enterohepatic *Helicobacter* species (Table S2). In a clustering analysis based on the pairwise ANI values (Fig. S1), the HCCM strains formed a cluster with the minimum within-cluster ANI values of 93.07 % and were clearly distinguished from the other enterohepatic *Helicobacter* species. *H. labetoulli* showed the highest ANI value to the HCCM cluster strains (87.6–88.84 %), and those of the other enterohepatic *Helicobacter* species were below 80.85 %.

**Fig. 1.**
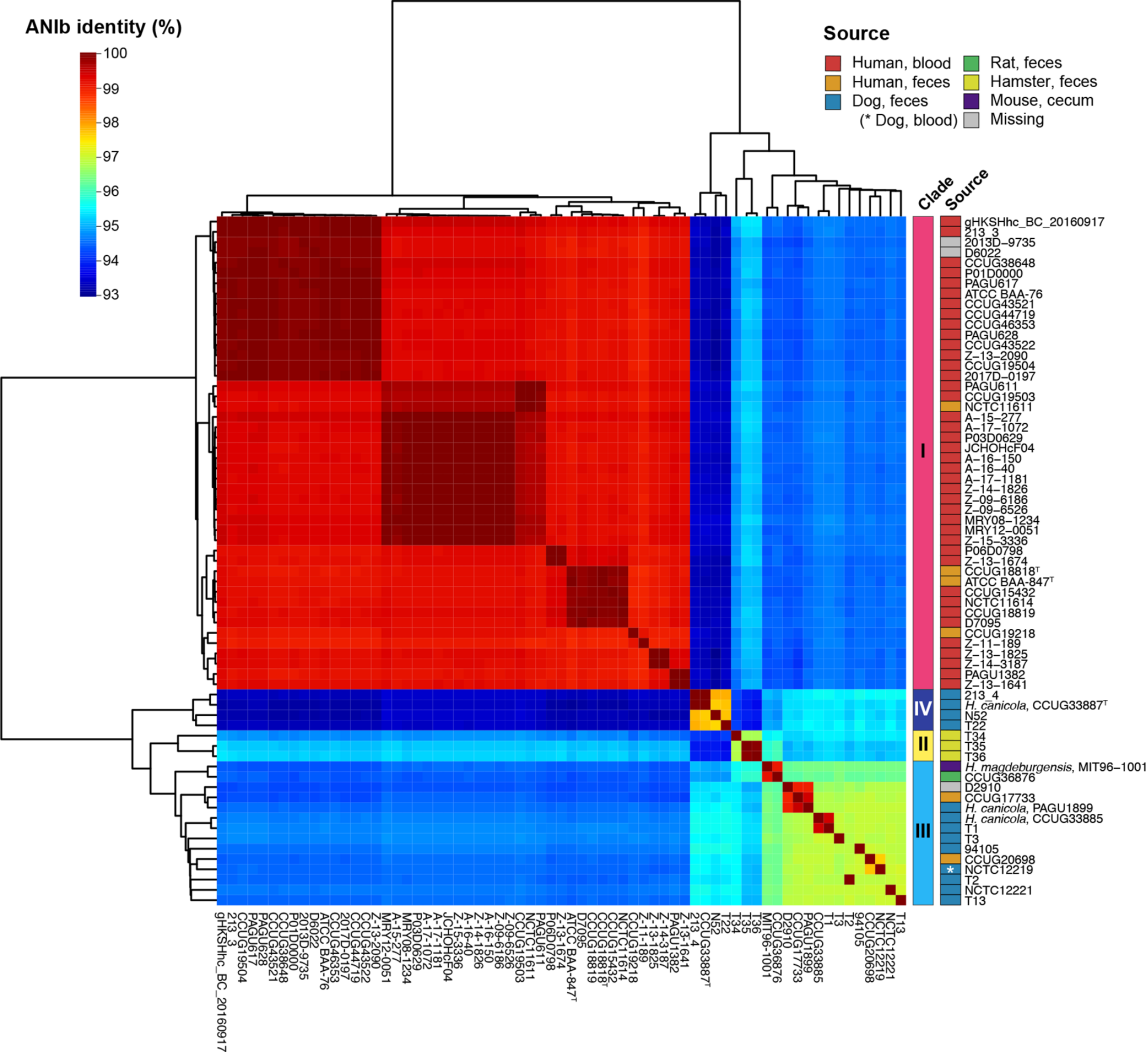
Pairwise ANI analysis of the 67 HCCM complex strains. ANI values were represented in a heatmap. Dendrograms were generated based on the results of average-linkage hierarchical clustering of the ANI values. Clades were defined using 96 % ANI as a cutoff. The isolation sources of each strain are shown.

Further analysis of the HCCM complex identified four distinct clades within the complex, named clades I–IV, with a 96 % ANI cutoff (Fig. 1, Table S6). The minimum within-clade ANI values of each clade were 98.8, 96.7, 96.1 and 97.6 %, respectively. The type strain of *H. cinaedi* (ATCC27087^T^) was included in the largest clade, clade I (*n*=46), and that of *H. canicola* (CCUG33887^T^) was included in clade IV, indicating that clades I and IV correspond to *H. cinaedi sensu stricto* and *H. canicola sensu stricto*, respectively.

‘*H. magdeburgensis*’ MIT96-100, the only strain whose genome sequence was deposited as ‘*H. magdeburgensis*’ [38], belonged to clade III, which also included two strains registered as *H. canicola* in the NCBI database.

### Core gene-based phylogenetic analysis of the HCCM strains

**Fig. 2.**
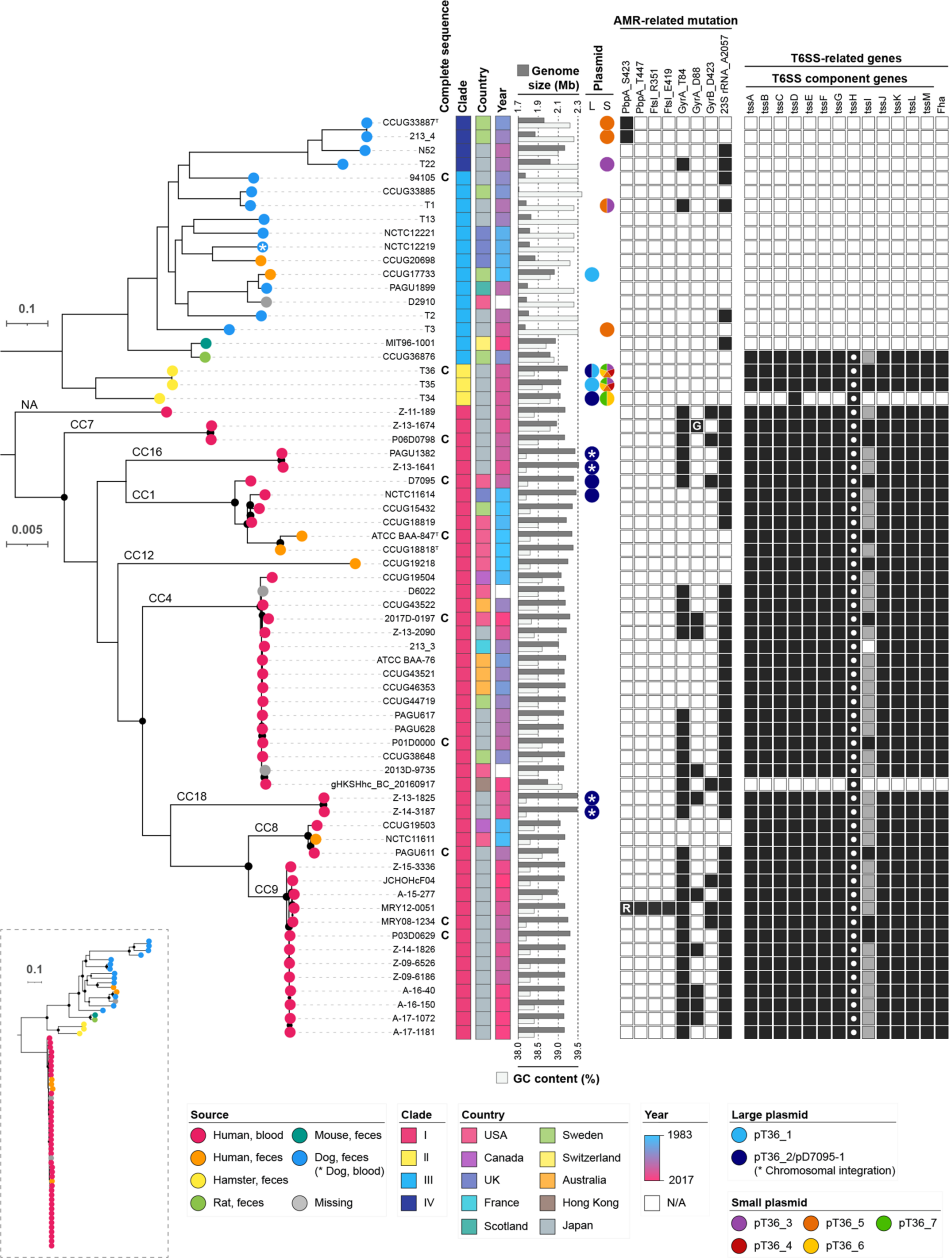
Phylogenetic relationships of the 67 HCCM complex strains. The core-gene-based ML tree of the 67 strains was constructed using the GTR+G4 model and 100 bootstrap replicates. The root of the HCCM complex strains, shown in a dashed box, was inferred from the phylogenetic relationship of the 67 strains with *H. labetoulli*, an enterohepatic *Helicobacter* most closely related to the HCCM complex (Fig. S1). The isolation source, clade, country, isolation year, genome size (Mb), GC content (%), and the presence of plasmids (*L*:≥5 kb, and *S*:<5 kb) are shown for each strain. Plasmid sequences that were integrated into chromosomes are indicated by asterisks. The presence of AMR-related mutations and T6SS genes are indicated by black squares. Capital letters (R and G) in black squares indicate substitutions by amino acid residues different from those in the known mutations. The presence of *tssI* genes was confirmed by tblastn search and is indicated by grey squares. The *tssH* gene (indicated by open circles) is located outside the HVRs shown in Fig. 6. Ten strains whose complete genome sequences are available are also indicated.

We identified core genes (*n*=1117) of the 67 HCCM isolates and *H. labetoulli* strain 48519 (used as an outgroup), and constructed a core-gene-based phylogenetic tree (dashed box in Fig. 2 and S2). This analysis revealed that the branch to *H. labetoulli* was located between clade I and the other clades and that, among clades II/III/IV, clade II separated first from the others, and clade IV finally emerged from clade III. This analysis also revealed that clade I strains were very closely related, reflecting the high pairwise ANI values of these strains. In a phylogenetic tree based on the core genes (*n*=1221) of the 67 HCCM strains, which were identified by the analysis of these HCCM strains only (Fig. 2), the grouping of the strains by BAPS clustering was completely consistent of the grouping obtained by ANI-based clustering, providing strong phylogenetic support for ANI-based clustering with a 96 % ANI cutoff.

### Characteristics of the four clades

Clade I included all 40 human blood isolates as well as four human faecal isolates. Although the origins of two isolates were unknown, no nonhuman isolates were included. In contrast, clade II comprised three hamster isolates, clade III comprised mainly animal isolates (nine dog, one mouse, one rat strain, two human faecal isolates and one of unknown origin) and clade IV comprised four dog isolates, including the *H. canicola *-type strain. Thus, notable host specificity was observed for the four clades in our strain set, but enough cations are required to interpret the current data, particularly that of clades II and IV, because only small numbers of isolates are included in these clades.

**Fig. 3.**
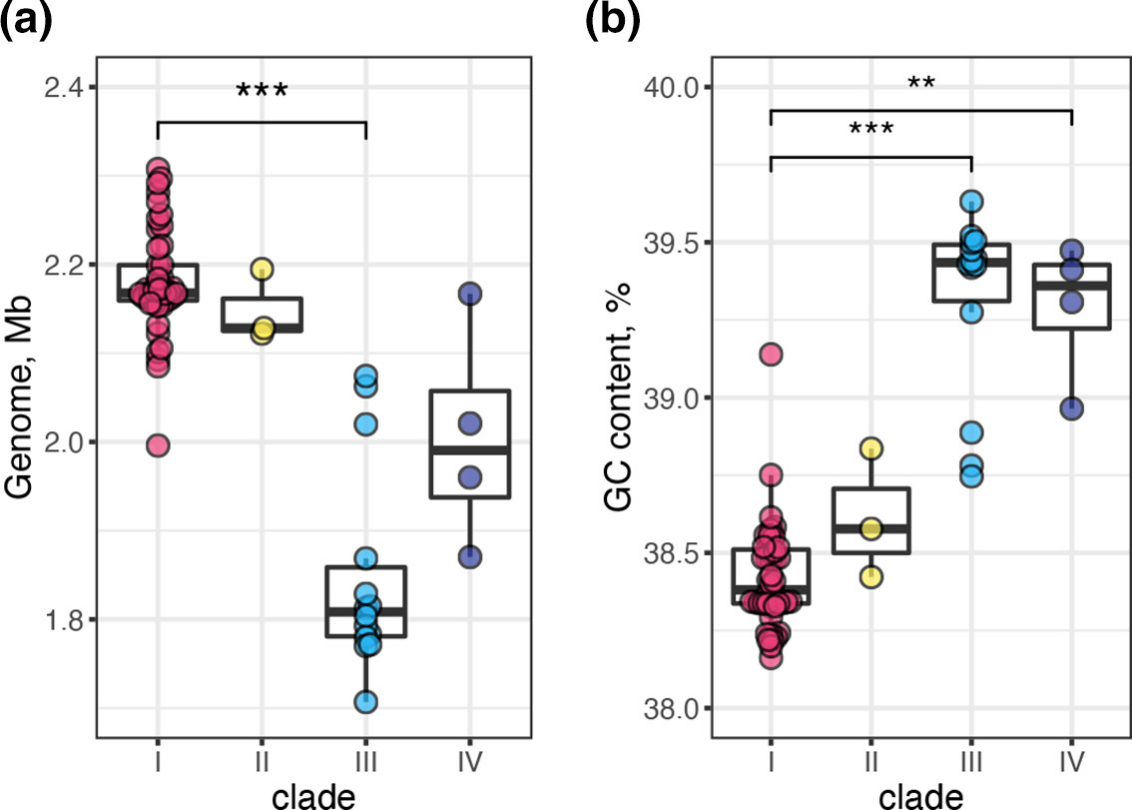
Differences in genome size (a) and GC content (b) between four clades. Student’s *t*-test was performed for statistical analysis (*P*<0.01; **, *P*<0.001; ***).

An interesting finding was the significant difference in genome size and GC content between the clades (Figs. 2 and 3). Clade I genomes (average, 2.18 Mb; range, 2.00–2.31 Mb) were significantly larger than clade III genomes (average, 1.85 Mb; range, 1.71–2.07 Mb; *P*-value, <0.001). The GC contents of clade I genomes (average, 38.42%; range, 38.16–39.14 %) were significantly lower than those of clade III (average, 39.32%; range, 38.74–39.63 %; *P*-value, <0.001) and clade IV (average, 39.29%; range, 38.96–39.47 %; *P*-value, <0.01) strains. The lack of HVRs that contain T6SS genes (see a later section) in most clade III (13/14) and all clade IV strains appears to partly contribute to the generation of these differences. The average size and GC content of clade II genomes were similar to those of clade I, but the differences compared to clades III and IV was not statistically validated due to the small number of clade II strains.

### Accumulation of AMR-related mutations in clade I

**Fig. 4.**
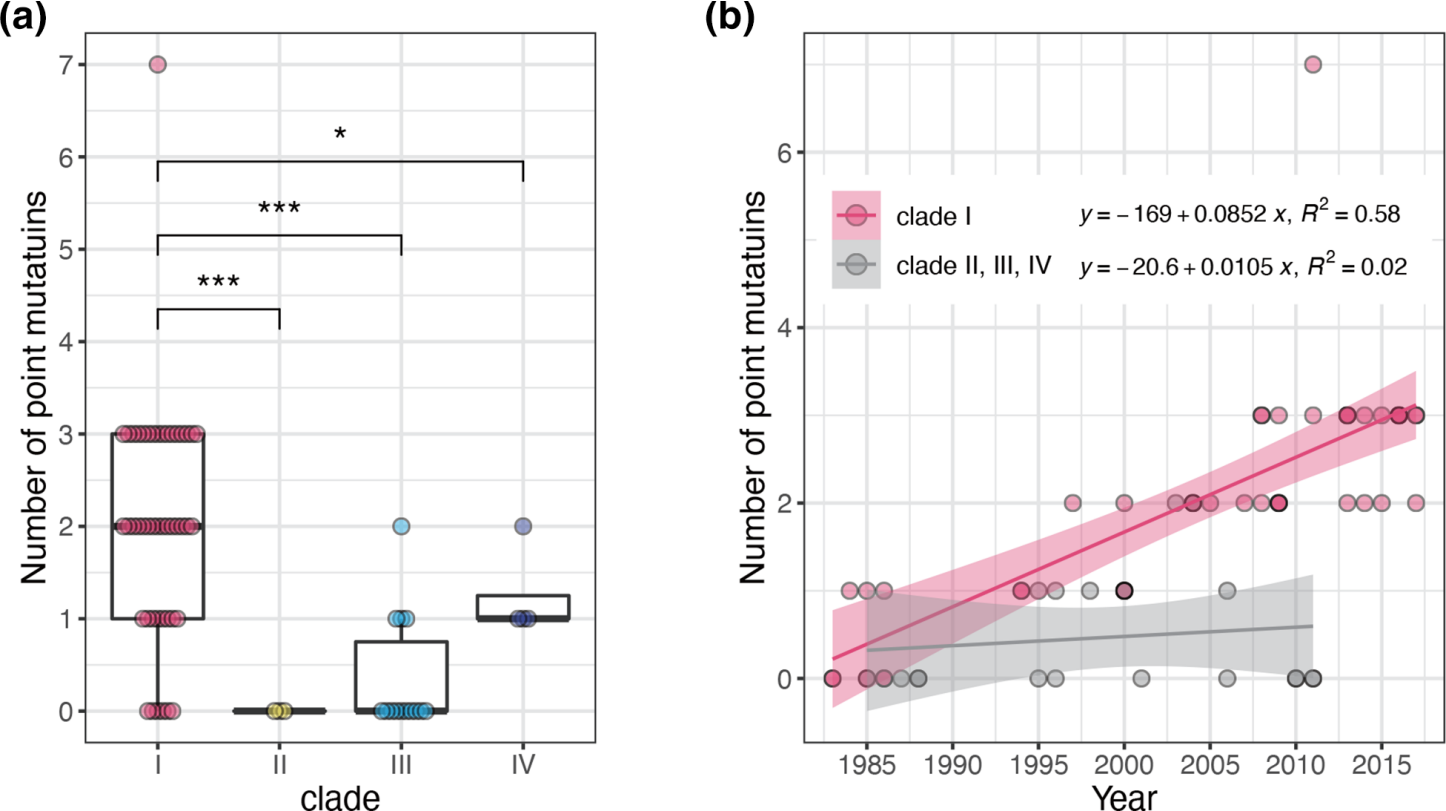
Accumulation of AMR-related point mutations. (a) Differences in the numbers of mutations between clades are shown. Student’s *t*-test was performed for statistical analysis (*P*<0.05; *, *P*<0.001; ***). (b) Correlation of the numbers of mutations and isolation years in clade I and the other clades (II/III/IV) is shown. The linear regression line, *R* ^2^ and 95 % confidence interval are indicated.

Many recent *H. cinaedi* clinical isolates have been reported to be resistant to quinolones and macrolides [13, 39]. The emergence of highly fluoroquinolone-resistant subclones during a hospital outbreak was also reported, which was associated with fluoroquinolone administration [5]. Although the presence of horizontally acquired AMR genes has not been shown for *H. cinaedi *, eight kinds of AMR-related point mutations have thus far been reported [5, 13, 26]. We therefore investigated the distribution of these mutations in our strain set (Figs. 2 and 4a). All eight mutations were detected among the clade I strains, and most clade I strains (40/46) contained one or more mutations with a median of two mutations (up to seven). In contrast, among the stains in the other clades, although three kinds of mutations were found in four clades III and all four clade IV strains, the numbers of mutations in these strains were one (6/8) or two (2/8) as shown in Figs. 2 and 4a. Thus, more AMR-related mutations have accumulated in clade I than in the other clades. In addition, more recent isolates contained more mutations (Fig. 4b), with an estimated accumulation rate of 1 mutation/10 years. We did not find any horizontally acquired AMR genes in our strain set.

The results of antimicrobial susceptibility tests of representative strains having various combinations of mutations confirmed that the strains carrying these mutations in the *gyrA* and 23S rRNA genes exhibited the resistance (or further increased resistance) to ciprofloxacin and erythromycin, respectively (Table S7). Although the strain carrying the mutations in *pbpA* and *ftsI* was not available in our laboratories, it was previously shown that the resistance to ceftriaxone is conferred by these mutations [13].

### Genes characteristic to clade I

Pangenome analysis of the 67 HCCM complex strains revealed a pangenome comprising 3887 genes. To further characterize clade I (*H. cinaedi sensu stricto*), we searched for the genes specifically enriched in clade I: those present in more than 80 % of the clade I strains and less than 20 % of the strains in the other clades (referred to as clade I-specific genes). The identified clade I-specific genes (*n*=227; Table S8) included genes related to a T6SS, a CRISPR-Cas system, type II toxin-antitoxin (TA) systems and various methyltransferase genes. All clade I strains encoded a class 2 type II-C CRISPR-Cas system, which was found only in two clade II strains and one clade III strain in the other clades (Table S9). As the presence of two CRISPR-Cas loci in the *H. cinaedi* chromosome was previously reported [40], we searched additional CRISPR-Cas loci using CRISPRCasTyper [30] and found another system similar to class 1 type III-D CRISPR-Cas in 55 strains belonging to clades I (*n*=42), II (*n*=2), III (*n*=8) or IV (*n*=3). However, a complete set of genes was not found in any of the strains. Among the methyltransferases identified, four were associated with restriction endonucleases, suggesting that they represent restriction-modification systems. Details of the T6SS are described in a later section.

We identified 12 genes that were absent in more than 80 % of the clade I strains and present in more than 80 % of the strains in the other clades, but most of them (8/12) encoded hypothetical proteins (Table S10).

### Plasmids, prophages and ICEs in the HCCM complex

Two plasmids were described in two *H. cinaedi* strains whose finished genome sequences were available (referred to as ‘completely sequenced strains’ in this manuscript): pHci1 (23 054 bp) in strain PAGU611 [11] and pD7095-1 (27 811 bp) in strain D7095 (GCF_014931105.1). We detected a *rep* gene in pD7095-1 but not in pHci1. Most pHci1 coding sequences were functionally unknown, and the pHci1 sequence showed notable similarity to a part of the chromosome sequences of strains ATCC BAA-847^T^ and P01D0000 (Fig. S3a). Thus, the status of pHci1 as a plasmid is currently unknown.

Among the other eight completely sequenced strains, we found plasmids only in a hamster isolate sequenced in this study (T36, clade II). This isolate contained as many as seven plasmids (Fig. S3b). Two were large plasmids, and the largest plasmid (pT36_1; 65 399 bp) was a linear plasmid, as revealed by FIGE analysis (Fig. S3c). The second largest plasmid, pT36_2 (27 822 bp), was highly homologous to pD7095-1 (≥96 % nucleotide sequence identity across the entire length; Fig. S3D). The remaining five plasmids (pT36_3~pT36_7) were smaller than 4 kb (Fig. S3b). Additional plasmids were not found in the search of draft genome sequences.

The distribution of the seven plasmids in the 67 HCCM complex strains was searched using the *rep* genes of each plasmid of strain T36 as markers (Fig. 2). These plasmids were found in two or more other strains (up to eight strains). While three small plasmids were found only in clade II, four (pT36_1, pT36_2/pD7095-1, pT36_3 and pT36_5) were found in multiple clades. pT36_2/pD7095-1-like plasmids were detected in six clade I strains and one additional clade II strain, suggesting transmission between clade I human isolates and clade II hamster isolates, which was probably mediated by a conjugation system encoded by pT36_2/pD7095-1 (Fig. S3b). While the transmission mechanisms of the other plasmids are unknown, four small plasmids (pT36_3, _4, _5 and _6) carried mobilization-related genes (Fig. S3b). Chromosomal integration of pT36_2/pD7095-1-like plasmids was also found in four clade I strains (indicated by asterisks in Fig. 2; an example is shown in Fig. S3d), but the integration mechanism is currently unknown.

We also identified one chromosome region (58 761 bp) flanked by *attL*/*attR*-like sequences in strain CCUG19218, but this region is an integrative-conjugative element (ICE) because it contains a set of type IV secretion/conjugation system genes and an integrase gene.

### Variable chromosome regions in the HCCM complex

**Fig. 5.**
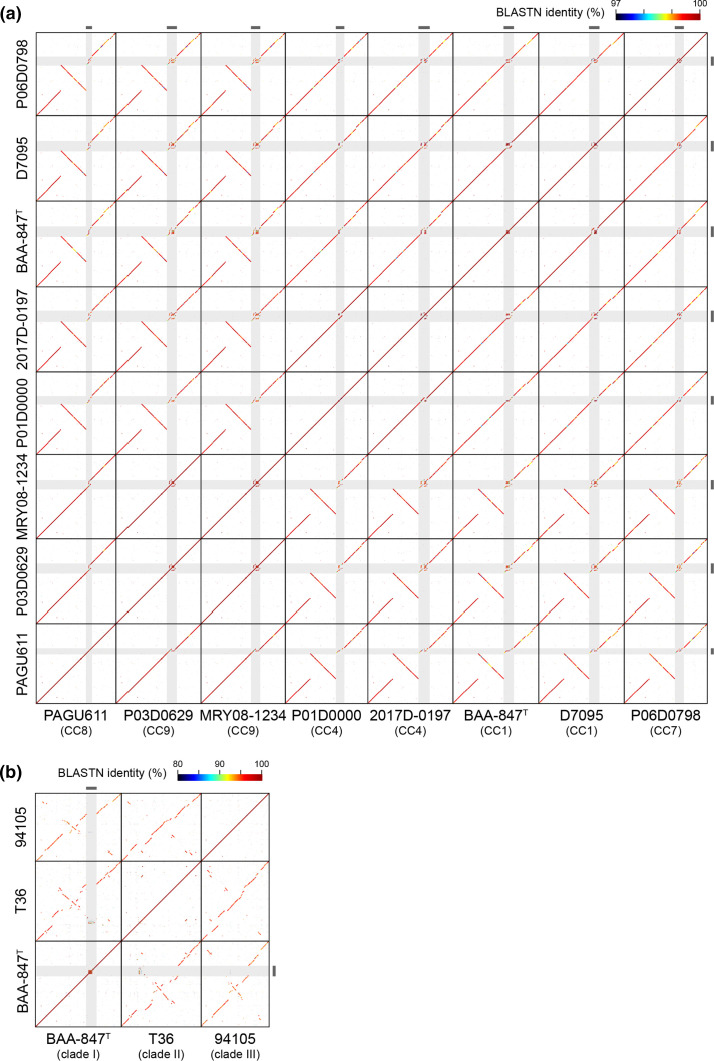
Dot plot matrix analysis of the chromosome sequences of completely sequenced HCCM complex strains. Highly variable regions (HVRs) are indicated by light grey shading and bold lines on the top and right-hand sides. (a) Comparison of eight clade I strains. (b) Comparison of a clade II strain (t36), a clade III (94105) strain and a clade I strain (ATCC BAA-847^T^).

Our strain set contained ten completely sequenced strains: eight in clade I, one in clade II and one in clade III (Table S1). The eight clade I strains belonged to four clonal complexes: CC1 (two strains), CC4 (two strains), CC7, CC8 and CC9 (two strains). Using these finished genome sequences, we analysed structural variations of chromosomes among the HCCM complex by dot plot analysis (Fig. 5a). The chromosomes of clade I strains showed well-conserved genomic synteny except for a large inversion that occurred in the CC8 and CC9 strains (corresponding to positions 656973–1 322 103 in ATCC BAA-847^T^). However, we found a region showing high sequence variation between the eight strains (indicated by grey shading in Fig. 5(a); referred to as a highly variable region, HVR). A set of T6SS genes were encoded in this region, but their gene organization and sequence were highly variable, as described in the next section.

Comparison of the chromosomes of clade II (hamster isolate, T36) and clade III (dog isolate, 94105) strains with the ATCC BAA-847^T^ chromosome revealed that although genome synteny was conserved between these strains to some extent, there were numerous deletions/insertions and inversions in the chromosomes of the clade II and clade III strains relative to that of ATCC BAA-847^T^ (Fig. 5b). Sequences homologous to parts of HRV of ATCC BAA-847^T^ were detected in the clade II strain but not in the clade III strain.

### HVRs and the T6SS encoded therein

**Fig. 6.**
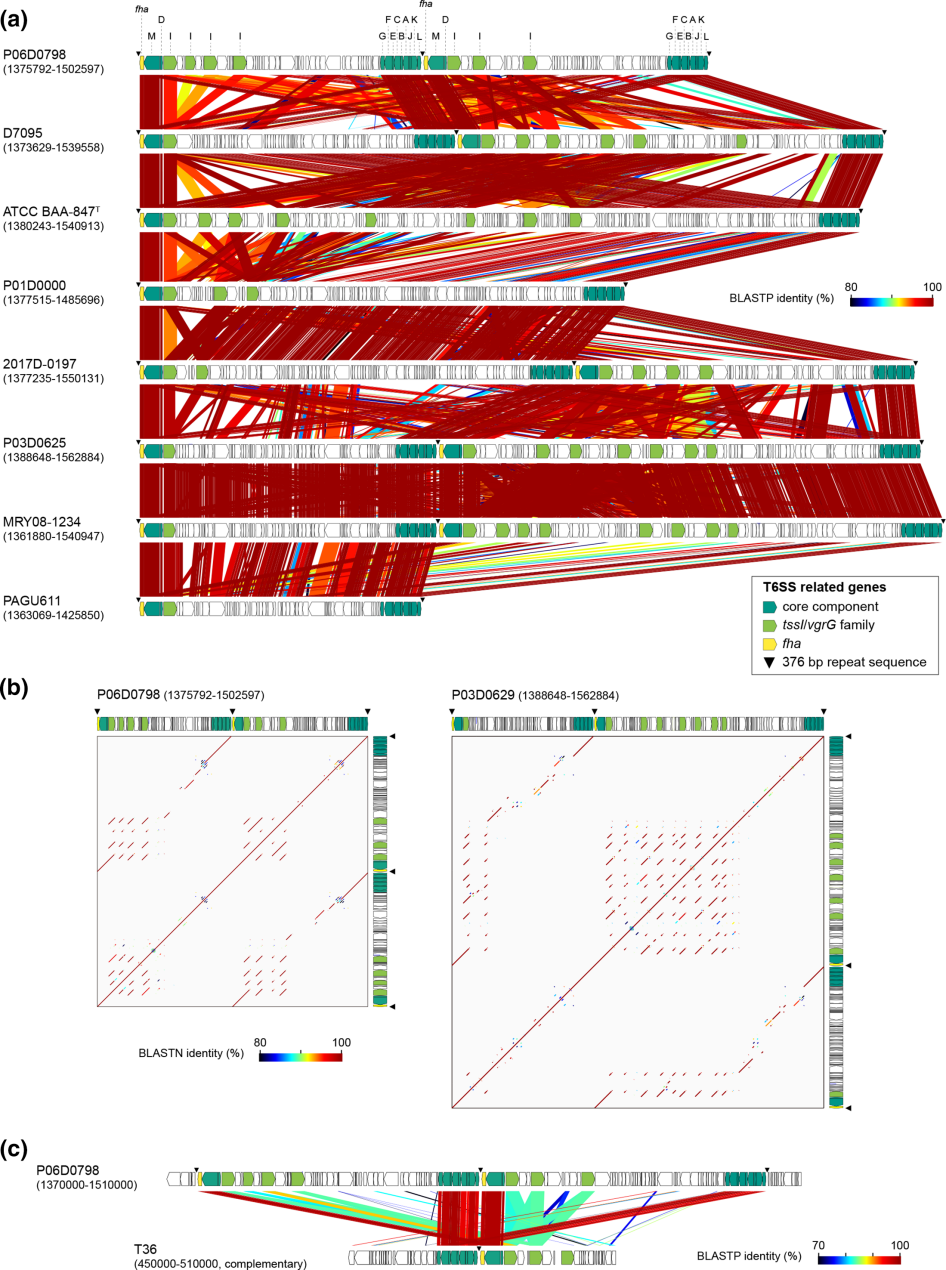
Comparison of highly variable regions (HVRs) containing T6SS-related genes between eight completely sequenced clade I strains. The gene organization in each HVR and amino acid sequence homologies between genes are shown. T6SS-related genes were divided into three groups and indicated by different colours. (a) Comparison of the HVRs of eight clade I strains. (b) Self-to-self dot plot analysis of the HVRs containing two sets of T6SS-related genes. The data of two strains (P06D7098 and P03D0629) are shown as examples. See Fig. S4 for the data of the remaining three strains. (c) Comparison of a genomic region of a clade II strain (T36) corresponding to the HVR in clade I and the HVR of a clade I strain (P06D0798).

The HVR of strain PAGU611 was included in the HciGI1 genomic island (131.1 kb) previously reported for this strain [11]. Detailed analysis of the HVRs of the eight clade I strains confirmed that the sequences and gene organizations of HVRs differed notably between clade I strains, even between the strains belonging to the same CC, and revealed that they encoded one or two sets of T6SS genes (Fig. 6a). In five strains that contained two sets, a set of 13 genes encoding TssD/M, Fha, TssI, and TssL/K/J/A/B/C/E/F/G was located in the middle of the HVRs, and the same set of genes with nearly identical sequences was separated into two subsets located at the left and right ends of each HVR. The same subsets of genes were located at the ends of HVRs in three strains that contained one set of T6SS genes. These proteins are components of the T6SS apparatus, except for Fha (Forkhead-associated protein), which is an accessory protein known to play a critical role in the posttranslational regulation of T6SS activity [41]. Although a homologue of TssH (another T6SS component) was encoded outside the HVR in all eight strains, TssH may not be essential for the function of T6SS of *H. cinaedi* because it has been shown that T6SS was functionally active even if TssH was absent in a species closely related to *Helicobacter, Campylobacter jejuni* [41]. In addition to these T6SS-related proteins, one or more TssI/VgrG family proteins (spike proteins) were encoded in the HVRs (up to nine copies).

Further analysis of the HVRs that encoded two sets of T6SS genes by dot plot analysis provided signs of duplication of the segments between of the two sets of T6SS genes and local duplications of *tssI* and its flanking genes (Figs. 6B and S4). As identical 376 bp sequences were present at the left and right ends of the HRV and between *tssL* and *fha* in the central T6SS gene set (Fig. 6)*,* this sequence may be involved in segment duplication, although accurate duplication mechanisms are currently unknown.

In the complete genome of the clade II strain (but not in the complete genome of the clade III strain), the same 13 T6SS genes and three copies of *tssI* genes were encoded at an analogous chromosome region (Fig. 6c). Rescreening of the T6SS genes (*tssA-L* and *fra*) in the draft genomes revealed that these genes were fully conserved in all but one clade I strain, an additional clade II strain, and one clade III rat strain (Fig. 2), but most clade III strains and all clade IV strains contained none of these genes. Although the analysis of *tssI* was complicated by the presence of multiple copies, we confirmed its presence in these T6SS-positive strains by tblastn search using the TssI protein sequences from finished genomes, except for one clade I strain (Fig. 2). In the clade I strain (gHKSHhc_BC_20160917) lacking all T6SS genes other than *tssH*, a deletion of a large segment including the HVR and flanking regions (corresponding to nucleotide positions 1 376 805–1 505 220 of the chromosome of P01D0000, a close relative of this strain) occurred (Fig. S5).

We performed a phylogenetic analysis of the *tssG-tssL* regions in the eight finished clade I genomes and two clade II and one clade III genomes that contained T6SS genes (Fig. S6). In this analysis, the *tssG-tssL* regions of clade I and those of clades II and III formed distinct clusters. While both clusters showed very high intracluster nucleotide sequence identities (over 99.48 and 99.99 %, respectively), intercluster sequence identities were also high (between 97.32 and 97.59 %), suggesting that the T6SS genes of the two clusters were derived from the common ancestor and their separation was a relatively recent event. In addition, this phylogenetic analysis revealed that the two sets of the *tssG-tssL* genes found in five clades I genomes were identical in sequence, respectively, supporting the occurrence of segment duplication of HVRs.

### Detection of clade I (*H. cinaedi sensu stricto*) in human faecal metagenomes

Our knowledge on the prevalence of *H. cinaedi* in the intestines of healthy individuals as well as the natures of *H. cinaedi* strains colonizing there is very limited. Only one report described the detection of *H. cinaedi* DNA by PCR in 13.3 %(4/30) of the Japanese healthy volunteers analysed [7]. Therefore, based on the genomic features revealed by this study, we performed a systematic search of the *H. cinaedi* genome sequences in the healthy human metagenome data available in the NCBI SRA database.

**Fig. 7.**
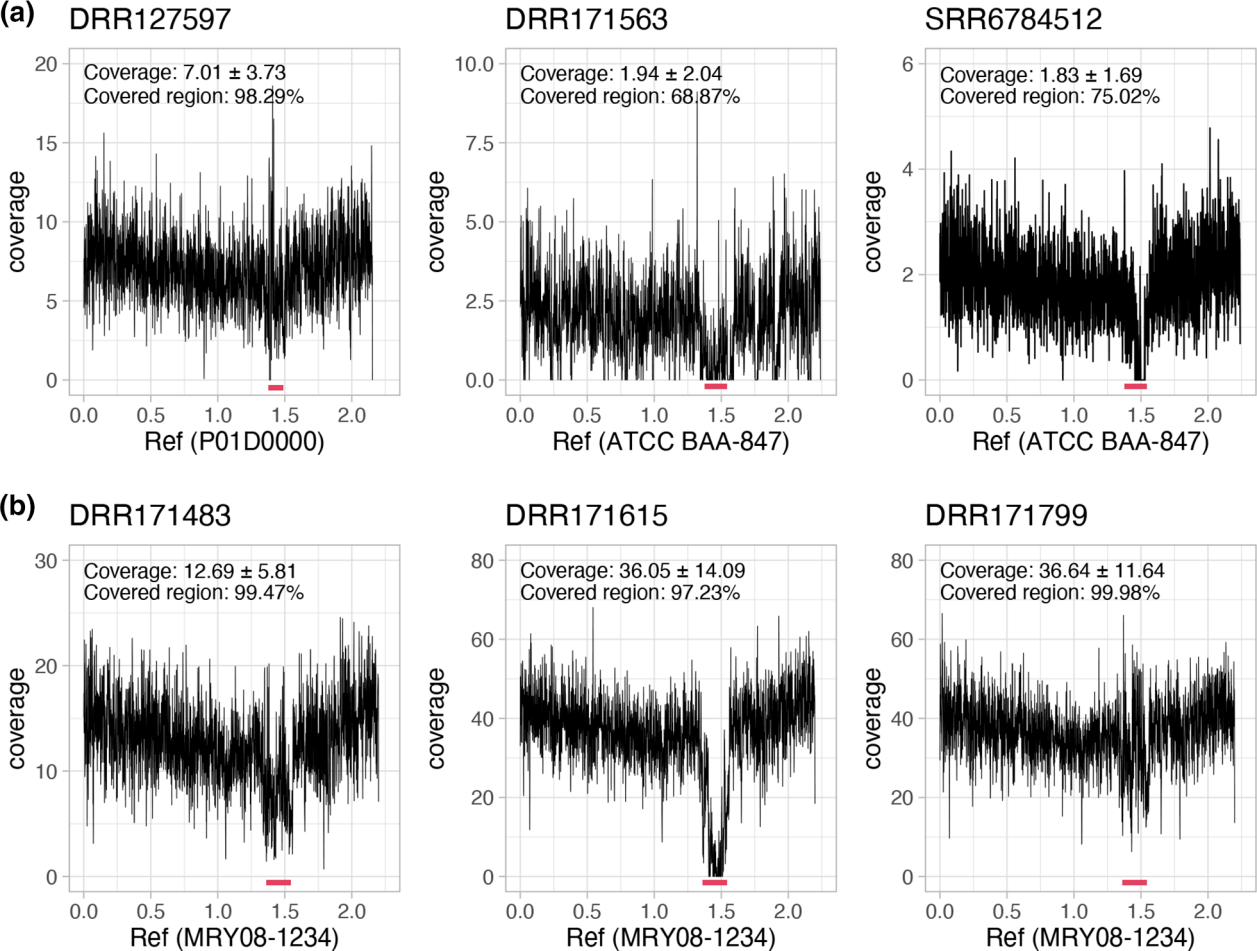
Genome sequences of clade I (*H. cinaedi sensu stricto*) strains detected in metagenome data from three healthy individuals (a) and three colorectal cancer patients (b). The results of read mapping to reference chromosome sequences are shown. The names of metagenome samples and references are indicated at the top and bottom of each panel, respectively. The HVRs are indicated by red lines.

For this purpose, we designed two sets of three 31-mer sequence probes, one to detect clade I and one to detect the HCCM complex (Table S4). The sequences of the former set of probes were conserved in clade I but absent in the other clades, and those of the latter were conserved in the entire HCCM complex. Using these probes, we searched 715 metagenome data (Table S5) and detected hits in three SRA datasets (from two Japanese individuals and one American individual) by two or three probes in both sets (Table 1), indicating that these three samples contained clade I strains. By determining their STs, we selected the complete genomes most closely related to each metagenome strain as references and mapped all reads from each SRA sample to the reference genomes. In this analysis, SRA reads were mapped to 68.80–98.29 % of the references, with a roughly even mapping depth except for the HVR (Fig. 7a), confirming the presence of clade I strains in these three healthy individuals as well as the sequence diversity of their HVRs. The prevalence of clade I (*H. cinaedi sensu stricto*) in all healthy individuals examined was 0.4%, and that in Japanese individuals was 1.13 %(3/265), which was lower than the result reported by Oyama K *et al.* [7].

**Table 1.** *
H. cinaedi *-containing human gut microbiome samples identified by using *H. cinaedi *- and HCCM complex-specific sequence probes

									MLST allelic profile (allele No.)											
Accessionno.	Bioproject	Country	Sex^*a*^	Age^*b*^	BMI	Appendix^*c*^	Probe hit	Sequence amount(Gb)	23SrRNA	*ppa*	*aspA*	*aroE*	*atpA*	*tkt*	*cdtB*	ST^*e*^	CC^*e*^	Reference sequence used for analysis	Sequence coverage (x)	Sequence coverageper Gb
DRR127597	PRJDB4176	Japan	F	53	18.7	Healthy	IPb1-3, CPb1-3	5.0	3	1	3	1	4	1	1	5	4	P01D0000	7.01	1.40
DRR171563	PRJDB4176	Japan	M	32	18.7	Healthy	IPb1/2, CPb1-3	5.3	1	1	1	1	1	1	1	1	1	ATCC BAA-847	1.94	0.37
SRR6784512	PRJNA379741	USA	M	n/d	31.3	Healthy	IPb1-3, CPb1/2	19.3	1	1	1	1	1	1	1	1	1	ATCC BAA-847	1.83	0.09
DRR171483	PRJDB4176	Japan	M	61	22.4	CRC, Stage_0	IPb1-3, CPb1/2	5.3	4	2	2	2	2	1	1*^d^*	10	9	MRY08-1234	12.69	2.39
DRR171615	PRJDB4176	Japan	F	57	19.4	MP	IPb1-3, CPb1-3	5.3	4	2	2	2	2	1	1^*d*^	9	9	MRY08-1234	36.05	6.80
DRR171799	PRJDB4176	Japan	M	70	21.0	CRC, Stage_I_II	IPb1/3, CPb1-3	4.9	4	2	2	2	2	1	1^*d*^	10	9	MRY08-1234	36.64	7.48

*a*, F, Female; M, male.

*b*, n/d; no data.

*c*, MP, multiple polypoid adenomas with low-grade dysplasia (more than three adenomas, mostly more than five adenomas); CRC, colorectal cancer. Appendix information of PRJDB4176 was referred to [Bibr R49][49].

*d*, One difference (97G -> 97T) found to allele no. 1.

*e*, ST, sequence type; CC, clonal complex.

We performed a similar analysis of the faecal metagenome data of nonhealthy Japanese individuals from two BioProjects (420 samples in total, Table S5) since many *H. cinaedi* infections have been reported in Japan in the current decade [42–44]. This analysis identified three colorectal cancer (CRC) patients carrying clade I strains (Table 1). In these cases, the SRA reads were mapped to more than 97 % of the reference genomes, and the mapping depth dropped at the HVRs (Fig. 7b). The prevalence of clade I in this analysis was 0.7 %. Although no statistically significant difference in the *Helicobacter* prevalence between the metagenomes of healthy people and CRC patients was detected (Fisher’s exact test), mapping depth normalized by the number of total sequence reads was higher for the strains in three CRC patients than for those in the three healthy individuals (Table 1).

## Discussion

In this study, we analysed the phylogenetic relationships and genetic diversity of the HCCM complex based on their genome sequences and obtained several important findings. First, the HCCM complex consisted of four clades (Figs. 1 and 2, S1 and S2). All human isolates belonged to clade I, which apparently corresponds to *H. cinaedi sensu stricto* because it included the type strain of this species*,* except for two human faecal isolates belonging to clade III. No nonhuman isolates were found in clade I. Thus, it is most likely that *H. cinaedi sensu stricto* represents a human-adapted lineage in the HCCM complex, which is a potentially pathogenic symbiont of human gut microbiota, so call pathobiont [45]. In contrast, clade IV, which corresponds to *H. canicola sensu stricto* because it included the type strain of *H. canicola *, is comprised of only dog isolates. Clade III was comprised of mainly dog isolates (nine of the 14 isolates; two were registered as *H. canicola* while the others were registered as *H. cinaedi *), but also included two human, one rat and one mouse faecal isolates, and a new species name ‘*H. magdeburgensis*’ was proposed for the mouse isolate although it has not been validated [38]. Of the two human faecal isolates in clade III, CCUG17733 was isolated from a 1-year-old baby, and CCUG20698 was isolated from a female child with diarrhoea [46]. Although premature or dysbiotic intestinal microbiota may facilitate colonization or infection by non-clade I strains, this finding may suggest a possibility that clade III strains can be transmitted from animals to humans under certain conditions because all other members of clade III are animal isolates (mostly from dogs), but more clade III strains need to be analysed to understand their directionality of transmission. Clade II included only hamster isolates but was comprised of only three isolates. Regarding the taxonomic statuses of the four clades in the HCCM complex, if a 96 % ANI value is used as a threshold, they are regarded as different species, but the ANI values between clades I and II, clades II and III, and clades III and IV were just below 95 % or over 95 % (Table S6). Therefore, the taxonomic statuses of clades II and III are currently unclear. Further analyses using more non-clade I strains are required to make a clear conclusion on their taxonomic statuses. The human pathogenicity of non-clade I strains, particularly that of clade III, also needs to be investigated and compared to that of clade I in the future.

Second, we identified several genomic features unique to clade I. Clade I strains contained larger genomes with lower GC content than the other clades (Fig. 2). Clade I-specific enrichment of the genes for a specific CRISPR-Cas system and multiple TA and restriction-modification systems may suggest a considerable contribution of horizontal gene transfer to the evolution and niche adaptation of clade I (Table S8). In this regard, the presence of T6SS genes in clade I is also intriguing. Very similar T6SS genes were present in two clade II strains and one clade III strain and these genes were absent in the other non-clade I strains (Fig. 2 and S6). This finding suggests a possibility that the T6SS genes were acquired by the common ancestor of the HCCM complex and they were deleted from these T6SS-negative strains, although other possibilities such as the transfer of the T6SS genes between clade I strains and clade II/III strains can not be excluded. The T6SS genes in clade I were encoded in the HVRs, which contained genes for multiple and variable TssI/VgrG family spike proteins along with highly conserved gene sets for T6SS components. The HVRs of clade I are larger and more complicated than those of clade II. Moreover, the HVRs were duplicated in many clade I strains (five of the eight complete genomes), promoting the further diversification of T6SS in clade I (Fig. 6 and S4). The physiological roles of the T6SS in clade I (*H. cinaedi sensu stricto*) are currently unknown. However, considering the functions of T6SS described for other Gram-negative bacteria [47], it is most likely that the T6SS of clade I also plays important roles in the competition between clade I strains and against strains in the other clades of the HCCM complex as well as other closely or distantly related bacteria. This competition may partly contribute to the colonization of clade I strains in the human intestine. In addition, the T6SSs of *C. jejuni* and *H. hepaticus *, both of which are closely related to *H. cinaedi *, have been shown to increase bacterial pathogenicity by enhancing inflammatory responses as well as influencing bacterial adhesion to host cells and subsequent invasion [47, 48]. Therefore, in terms of both colonization in the human intestine and bacterial pathogenicity, functional analyses of the T6SS of *H. cinaedi* will be important in future studies of this bacterium.

Another important finding was the identification of seven plasmids, including a large linear plasmid (Fig. 2 and S3). An ICE was also identified for the first time in the HCCM complex, expanding our understanding of mobile genetic elements in this group of bacteria. The distribution of the plasmids in different clades and the presence of conjugation- and mobilization-related genes in many of the identified plasmids suggest the possibility of interclade transmission. Since ecological barriers may be present between clades, as suggested by the observed host specificity, it would be interesting to know how and where the interclade transmission of these plasmids occurs.

Finally, we developed a method useful for the systematic search of *H. cinaedi sensu stricto* and other HCCM complex members in metagenome data based on the genome sequence information obtained in this study. Using two sets of sequence probes, we successfully detected six HCCM complex-containing faecal metagenome samples from healthy individuals and CRC patients (Table 1). All six samples contained clade I strains, supporting our conclusion that *H. cinaedi sensu stricto* is a human-adapted lineage in the HCCM complex in terms of human commensal as well as a human pathogen. Furthermore, our read mapping data indicated that sequence information covering almost entire *H. cinaedi* genomes can be obtained if appropriate reference genomes are used and the sample contains *H. cinaedi *-derived reads corresponding to more than ×7 coverage (Fig. 7). The presence of HVRs could also be detected. Although our detection rates were lower than that in a previously reported PCR-based screening of healthy Japanese volunteers [7], the sensitivity of our method could be dependent on the amount of total sequence data and the relative amount of *H. cinaedi* reads. Despite these limitations, considering the difficulty of isolating *H. cinaedi* from faecal samples, the method we developed would be useful in analysing *H. cinaedi* and HCCM complex strains in faecal metagenome samples at the level of WGS. An interesting observation in our metagenome data analyses was the difference in the number of *H. cinaedi* reads between the samples from healthy individuals and CRC patients. Even after normalization by the amount of total reads, the samples from CRC patients contained notably more *H. cinaedi* reads than those from healthy individuals (Table 1). This difference may be due to technical problems related to sample or sequence library preparation, but it is also possible that the *H. cinaedi* population expanded in the intestine of CRC patients due to some dysbiosis induced in their intestinal microbiota. Further studies using more samples are required to examine whether the *H. cinaedi* population in the intestine expands under certain dysbiotic conditions.

In conclusion, through genome analysis of the HCCM complex, we identified four clades in the complex and found that clade I corresponds to *H. cinaedi sensu stricto* and represents a human-adapted lineage in the complex. Genomic features unique to clade I were also identified. Based on this genomic information, a method for analysing the genome sequences of *H. cinaedi sensu stricto* strains in the metagenome data was developed. These results provide not only an important basis for reconsidering the classification of the HCCM complex but also novel insights into the biology and ecology of the HCCM complex, particularly the evolution and host adaptation of *H. cinaedi sensu stricto*.

## Supplementary Data

Supplementary material 1

Supplementary material 2
