# Supplementary material for: Helicobacter cinaedi is a human-adapted lineage in the Helicobacter cinaedi/canicola/‘magdeburgensis’ complex
Source: Microb Genom. 2022 May 10;8(5):mgen000830. doi: 10.1099/mgen.0.000830 (PMC9465070; doi:10.1099/mgen.0.000830)
Supplement: Supplementary material 1 [file mgen-8-830-s001.pdf]

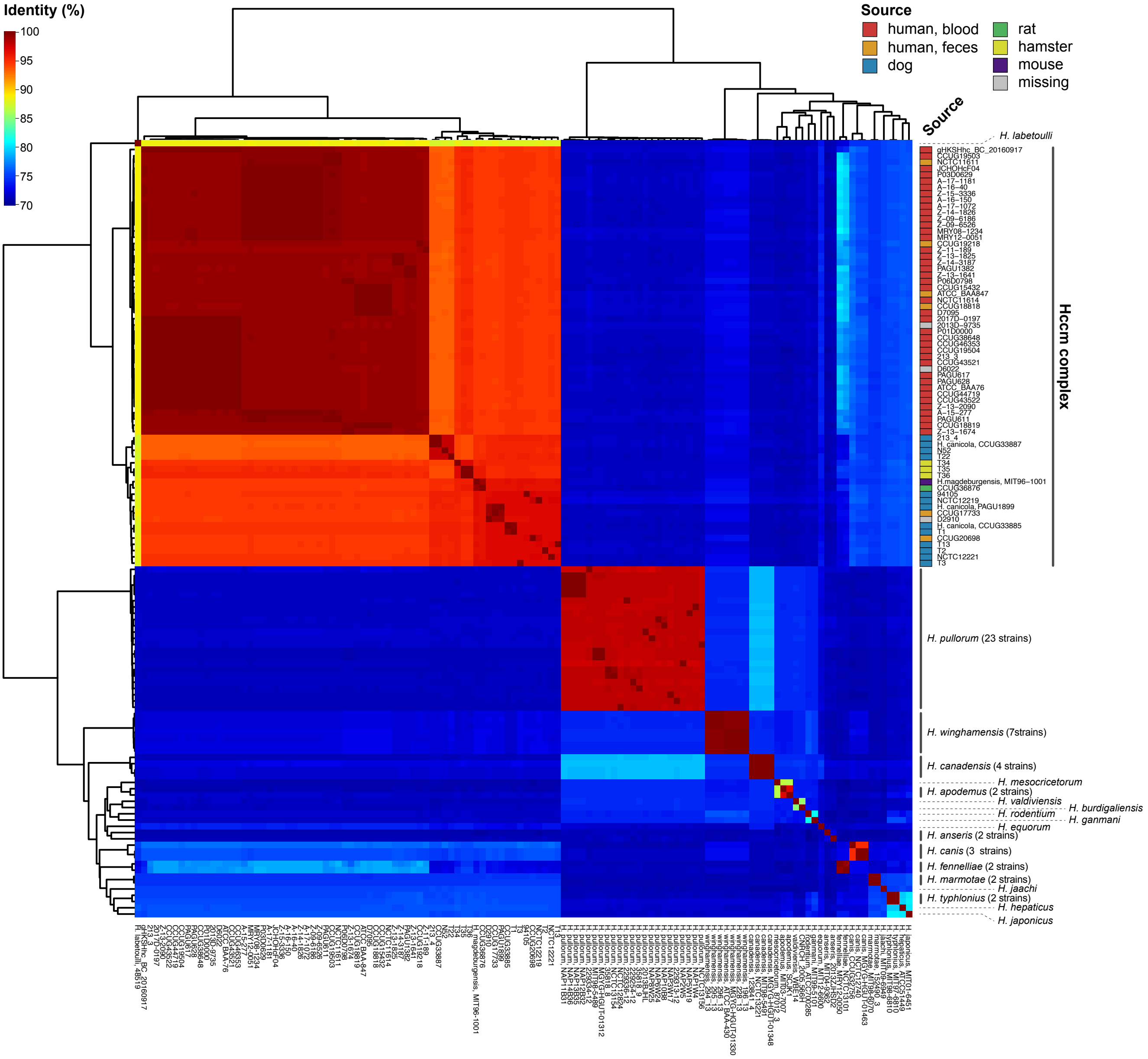

Fig. S1. Pairwise ANI analysis of strains belonging to the HCCM complex and other enterohepatic *Helicobacter* species.

ANI values are presented in a heatmap. Dendrograms were constructed based on the results of average-linkage hierarchical clustering of the values. The isolation sources of HCCM complex strains are shown.

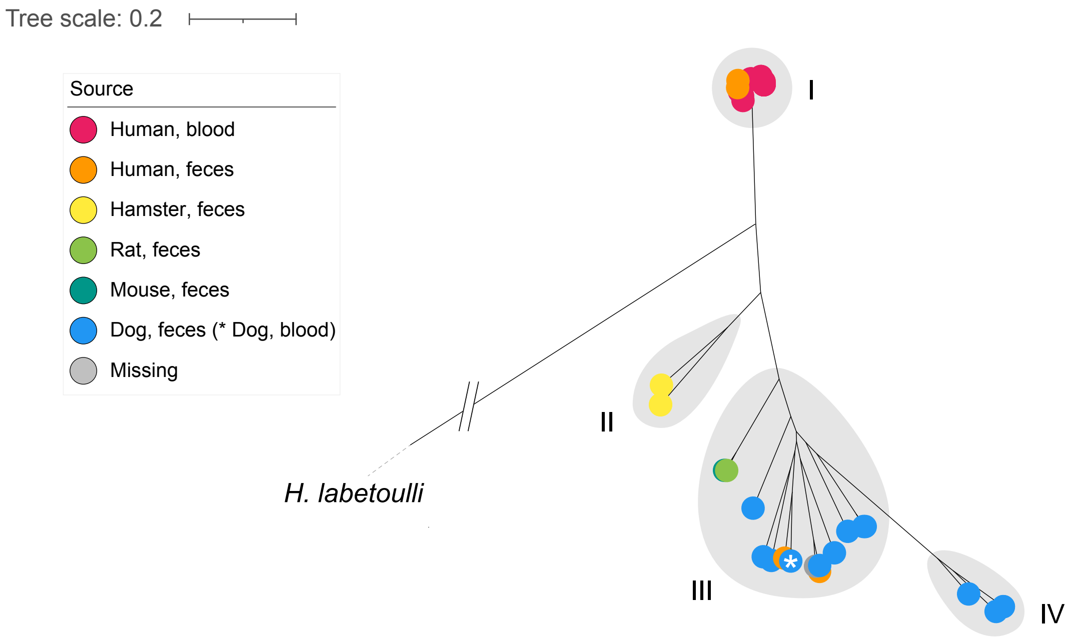

Fig. S2. Phylogenetic analysis of the 67 HCCM complex strains using *H. labetoulli* as an outgroup. An ML tree was constructed based on sequences of 1117 core genes using the GTR+G4 model and 100 bootstrap replicates. The results of pairwise ANI analysis shown in Fig. S1 indicate that *H. labetoulli* is the enterohepatic *Helicobacter* species most closely related to the HCCM complex.

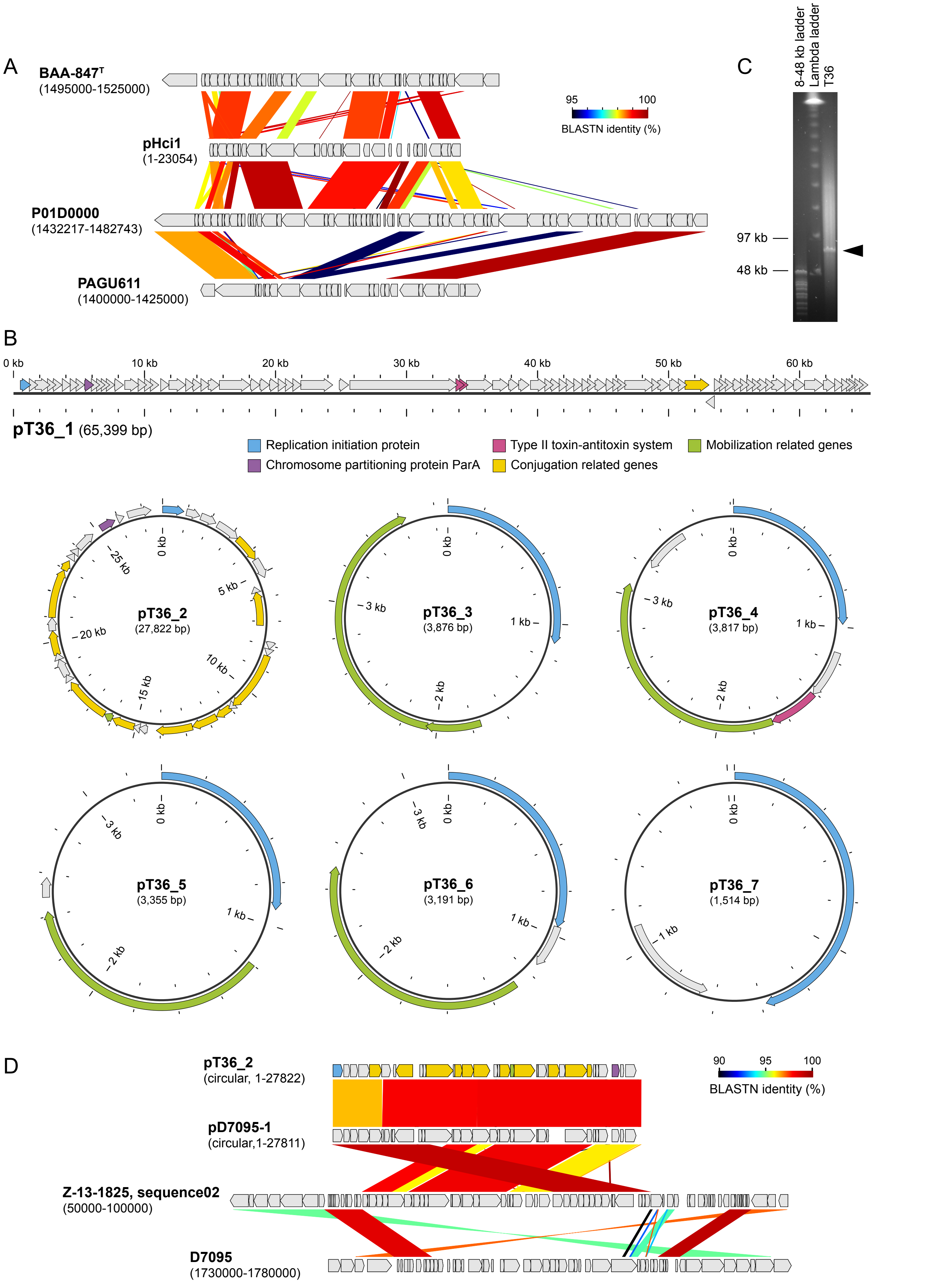

Fig. S3. Plasmid sequences found in this study.
(A) The nucleotide sequence of pHci1 showed notable similarity to part of the chromosome sequences of strains ATCC BAA-847^T and P01D0000. (B) Gene organization and functional annotation of seven plasmids identified in strain T36. (C) Field inversion gel electrophoresis analysis of total DNA purified from the T36 culture. The DNA band corresponding to the linear plasmid pT36_1 is indicated by an arrowhead. (D) Integration of a pT36_2-related plasmid in the chromosome of strain Z-13-1825.

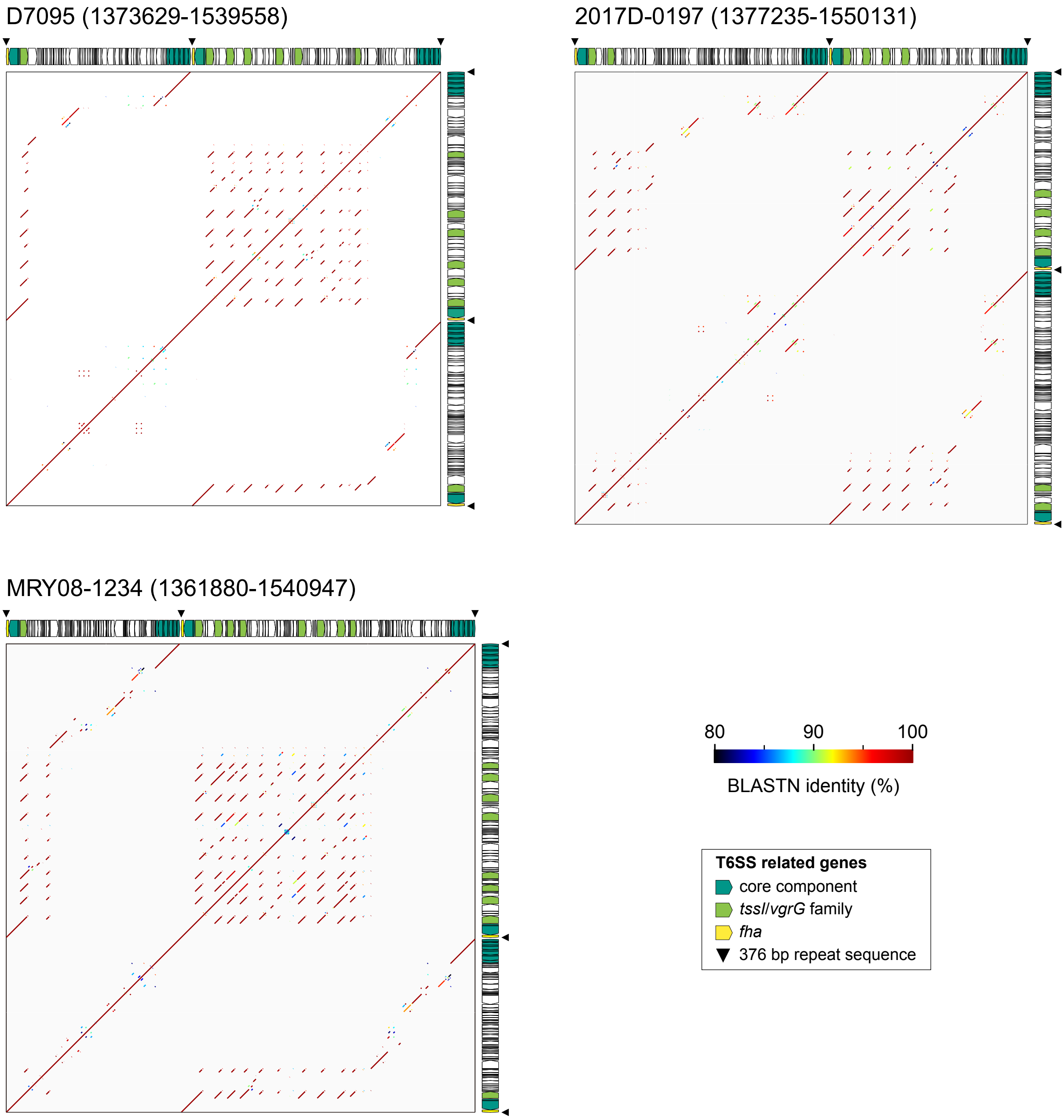

Fig. S4. Self-to-self dot plot analysis of the HVRs containing two sets of T6SS-related genes. The data of strains D7095, 2017D-0197, and MRY08-1234 are shown. See Fig. 6C for the data of the remaining two strains.

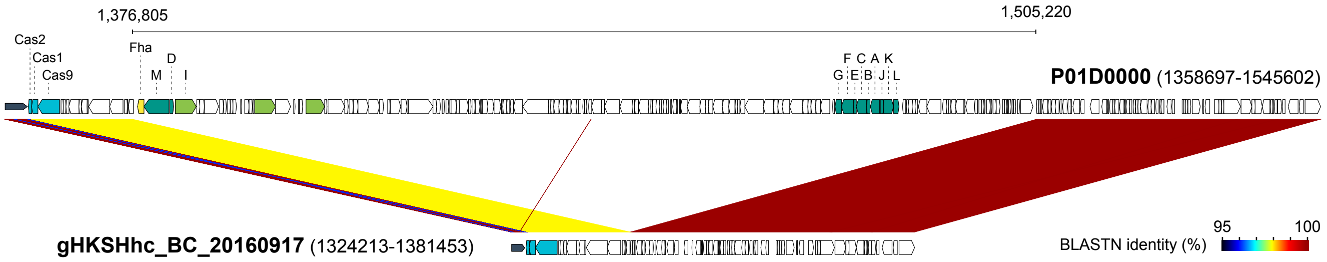

Fig. S5. The deletion of HVR in strain gHKSHhc_BC_20160917.
The chromosome region of strain gHKSHhc_BC_20160917 corresponding to the HVRs was compared with the HVR and its flanking regions of strain P01D0000.

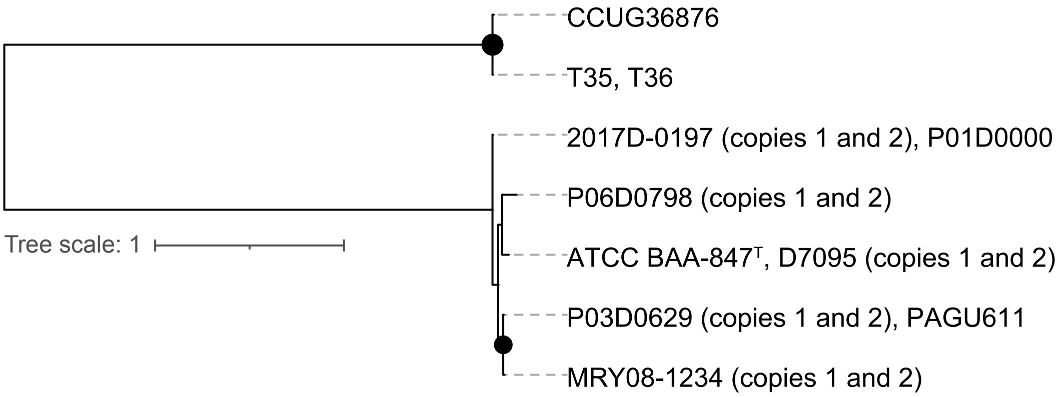

Fig. S6. Phylogenetic tree of T6SS sequences.
Recombination-free phylogenetic tree of T6SS sequences from *tssG* to *tssL* constructed using RAxML-NG ver. 1.0.1 and 1000 bootstrap replicates with the TIM1ef model. The sequences of the genome region of eight clade I finished genomes and two clade II and one clade III genomes that contained these T6SS genes were analyzed.
